# A novel coupled rainfall prediction model based on stepwise decomposition technique

**DOI:** 10.1038/s41598-024-61855-0

**Published:** 2024-05-13

**Authors:** Xueran Jiao, Zongheng He

**Affiliations:** Henan Key Laboratory of Water Pollution Control and Rehabilitation, Henan University of Urban Construction, Pingdingshan, 467000 China

**Keywords:** Stepwise decomposition technique, Bidirectional long short-term memory neural network, Improved particle swarm optimization, Variational mode decomposition, Rainfall, Environmental impact, Climate change, Hydrology

## Abstract

The traditional decomposed ensemble prediction model decomposes the entire rainfall sequence into several sub-sequences, dividing them into training and testing periods for modeling. During sample construction, future information is erroneously mixed into the training data, making it challenging to apply in practical rainfall forecasting. This paper proposes a novel stepwise decomposed ensemble coupling model, realized through variational mode decomposition (VMD) and bidirectional long short-term memory neural network (BiLSTM) models. Model parameters are optimized using an improved particle swarm optimization (IPSO). The performance of the model was evaluated using rainfall data from the Southern Four Lakes basin. The results indicate that: (1) Compared to the PSO algorithm, the IPSO algorithm-coupled model shows a minimum decrease of 2.70% in MAE and at least 2.62% in RMSE across the four cities in the Southern Four Lakes basin; the IPSO algorithm results in a minimum decrease of 25.58% in MAE and at least 28.19% in RMSE for the VMD-BiLSTM model. (2) When compared to IPSO-BiLSTM, the VMD-IPSO-BiLSTM based on the stepwise decomposition technique exhibits a minimum decrease of 26.54% in MAE and at least 34.16% in RMSE. (3) The NSE for the testing period of the VMD-IPSO-BiLSTM model in each city surpasses 0.88, indicating higher prediction accuracy and providing new insights for optimizing rainfall forecasting.

## Introduction

In recent years, the frequent occurrence of extreme rainfall events has posed a significant threat to socioeconomic well-being and human safety. Accurate simulation of rainfall processes holds crucial significance for water resources management^1,2^. The formation of precipitation is influenced by multiple factors, exhibiting a high degree of complexity and uncertainty^3,4^. Existing rainfall prediction models can generally be classified into two main categories: process-driven and data-driven^5^. The latter, data-driven models, operate without the need to consider the physical mechanisms underlying runoff occurrence. Instead, they solely entail mathematical analysis of time series data to establish functional relationships between input and output variables. Consequently, these models exhibit greater operational feasibility^6–8^. With the development of artificial intelligence and big data in recent years, the application of machine learning has presented new opportunities for rainfall prediction^9,10^. The Bidirectional Long Short-Term Memory Neural Network (BiLSTM) model, characterized by its simple structure, strong fault tolerance, and ability to capture long-term dependencies, has achieved successful applications in numerous rainfall prediction studies^11–13^. However, the predictive capability of a single model is limited. Coupling machine learning models with data decomposition methods such as ensemble empirical mode decomposition, wavelet decomposition, and variational mode decomposition (VMD) to establish decomposition ensemble models can effectively enhance model accuracy^14^. Among these methods, VMD demonstrates the ability to control central frequency aliasing phenomena and noise levels, making it easier to improve the predictive performance of decomposition ensemble models^15^.

Establishing coupled forecasting models to enhance rainfall prediction accuracy has emerged as a current research focus^16–18^. However, traditional decomposition methods typically involve initially decomposing the entire rainfall sequence and then partitioning the decomposed sub-sequences into training and testing periods. This approach of decomposition before partitioning leads to the premature utilization of testing period data prior to model training, which falls short of meeting actual forecasting requirements. Comparative analyses conducted by Du et al.^19^ and Wei et al.^20^ scrutinized the outcomes of various ensemble hydrological prediction models employing decomposition. They discovered that treating forecast factor data from the testing period as known information for constructing ensemble decomposition models results in "false" high-precision prediction outcomes. Some scholars have proposed stepwise decomposition techniques, wherein the observed sequence is initially divided into training and testing periods, followed by decomposition modeling of the training period, thus preventing the incorporation of future information into the training samples. Models developed based on stepwise decomposition techniques demonstrate reliable performance^21,22^. Wei et al. introduced a stepwise decomposition sampling technique to construct accurate decomposition ensemble models, aiming to enhance the prediction accuracy of correctly decomposed ensemble models^20^.

This study utilizes weekly rainfall data from the Nansi Lake Basin and employs a stepwise decomposition technique combined with variational mode decomposition (VMD) to construct training and testing sets for rainfall prediction models. A VMD-BiLSTM coupled model is developed to forecast the weekly rainfall sequence in the Nansi Lake Basin, with model parameters optimized using an improved particle swarm optimization (IPSO) algorithm. This model effectively addresses the issue of traditional ensemble decomposition models incorporating future information, thereby enhancing the prediction accuracy of decomposition ensemble models.

## Models and methods

### Variational mode decomposition

The variational mode decomposition (VMD)^23^ method is an innovative, fully non-recursive data decomposition approach that is adaptive in nature. This method achieves the decomposition of the original signal x into a series of Intrinsic Mode Functions (IMFs) by seeking the optimal solution to a constrained variational problem.

1 $$ \left\{ {\begin{array}{*{20}l} {\frac{\min }{{\left\{ {\mu_{k} ,\omega_{k} } \right\}}}\sum\limits_{k = 1}^{k} {\left\| {\partial_{t} \left[ {\left( {\delta \left( t \right) + \frac{j}{\pi t}} \right) \otimes \mu_{k} \left( t \right)} \right]e^{{ - j\varpi k^{t} }} } \right\|_{2}^{2} } } \hfill \\ {\sum\limits_{k = 1}^{k} {\mu_{k} = x} } \hfill \\ \end{array} } \right., $$

where $k$ represents the number of $IMF_{S}$; $\left\{ {\mu_{K} (t)} \right\} = \left\{ {\mu_{1} ,\mu_{2} , \cdots ,\mu_{k} } \right\}$ denotes the $kth$ modal component; $\mu_{k} \left( t \right)$ is the value of the $kth$ modal component at time $t$; $\left\{ {\omega_{k} } \right\} = \left\{ {\omega_{1} ,\omega_{2} , \cdots ,\omega_{k} } \right\}$ corresponds to the central frequency of the $kth$ modal component; $t$ stands for time; $\partial_{t}$ is the first-order derivative of the function with respect to time $t$; $\delta (t)$ is the unit impulse function; $j$ represents the imaginary unit; $\otimes$ signifies the convolution operation.

2 $$ \begin{gathered} L(\left\{ {\mu_{k} } \right\},\left\{ {\omega_{k} } \right\},\lambda ) = \alpha \sum\limits_{k = 1}^{k} {\left\| {\partial_{t} \left[ {\left( {\delta \left( t \right) + \frac{j}{\pi t}} \right) \otimes \mu_{k} \left( t \right)} \right]e^{{ - j\varpi k^{t} }} } \right\|_{2}^{2} } + \hfill \\ \left\| {x(t) - \sum\limits_{k = 1}^{k} {\mu_{k} (t)} } \right\|_{2}^{2} + \left\langle {\lambda (t),x(t) - \sum\limits_{k = 1}^{k} {\mu_{k} (t)} } \right\rangle , \hfill \\ \end{gathered} $$

where $\alpha$ represents the quadratic penalty factor; $\lambda$ denotes the Lagrange multiplier. $\lambda \left( t \right)$ is the value of $\lambda$ at time $t$, and $x\left( t \right)$ is the value of $x$ at time $t$. The alternating direction multiplier iteration algorithm is employed to solve the saddle point of Eq. (2).

### Improved particle swarm optimization

Due to the fact that in the basic particle swarm optimization algorithm, parameters $\omega$、$c_{1}$、$c_{2}$ are constants, the optimization process is highly susceptible to getting trapped in local optima, and its optimization capability is relatively poor when dealing with multiple objective functions and constraints. Therefore, an optimized improvement is proposed for the basic particle swarm optimization algorithm, aiming to make it more suitable for multi-objective problem solving.

3 $$ \left\{ {\begin{array}{*{20}l} {\omega^{\prime} = \omega_{\min } + \left( {\omega_{\max } - \omega_{\min } } \right)\left( {\frac{{t_{cur} }}{{t_{\max } }}} \right)^{2} } \hfill \\ \begin{gathered} c^{\prime}_{1} = c_{1i} + (c_{1f} - c_{1i} )\sqrt {\frac{{t_{cur} }}{{t_{\max } }}} \hfill \\ c^{\prime}_{2} = c_{2i} + (c_{2f} - c_{2i} )\left( {\frac{{t_{cur} }}{{t_{\max } }}} \right)^{2} \hfill \\ \end{gathered} \hfill \\ \end{array} } \right., $$

where $\omega^{\prime}$ is the improved inertia weight factor, with $\omega_{\max }$ set to 0.9 and $\omega_{\min }$ to 0.2; $c^{\prime}_{1}$ and $c^{\prime}_{2}$ are the refined learning factors; $t_{cur}$ represents the current generation count; $t_{\max }$ is the maximum number of iterations; $c_{1f}$ and $c_{2f}$ are the termination values for $c_{1}$ and $c_{2}$, set to 0.5 and 2, respectively; $c_{1i}$ and $c_{2i}$ are the initial values, taken as 2 and 0.5, respectively.

4 $$ \overline{P} = \frac{1}{N}\sum\limits_{i = 1}^{N} {P_{ij}^{t} } , $$

where $\overline{P}$ is the average of the optimal values of all individual particles; $N$ is the number of particles; $P_{ij}^{t}$ is the location of the optimal values of individual particles. The improved expression of the algorithm is:

5 $$ \upsilon_{ij}^{t + 1} = \omega^{\prime}\upsilon_{ij}^{t} + c^{\prime}_{1} r_{1} (\overline{P} - x_{ij}^{t} ) + c^{\prime}_{2} r_{2} (P_{gj}^{t} - x_{ij}^{t} ), $$

where $\upsilon_{ij}^{t + 1}$ is the velocity of the particle; $t$ is the number of selected generations; $r_{1}$, $r_{2}$ are random numbers in the interval [0–1], $x_{ij}^{t}$ is the position of the particle for $t$ iterations; $P_{gj}^{t}$ is the current optimal value position of all particles of the population.

### Bidirectional long short-term memory neural network

**Figure 1:**
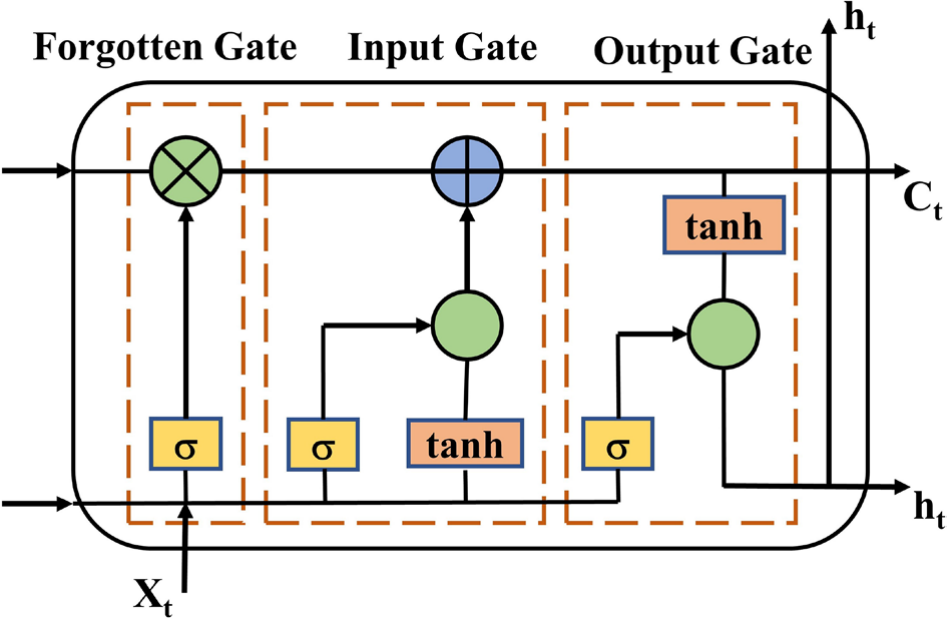
Structure of LSTM unit.

LSTM^24^ is a deep neural network that can accurately and efficiently learn long-term dependent information by introducing a gating mechanism that allows the model to selectively retain the function of transmitting long-term timing data information^25^. As shown in Fig. 1, it consists of three gates, input gate, output gate and forgetting gate and one core computing node. The forgetting gate, the input gate, and the output gate jointly realise the control to the unit state, selectively adding or removing information to the unit state.

**Figure 2:**
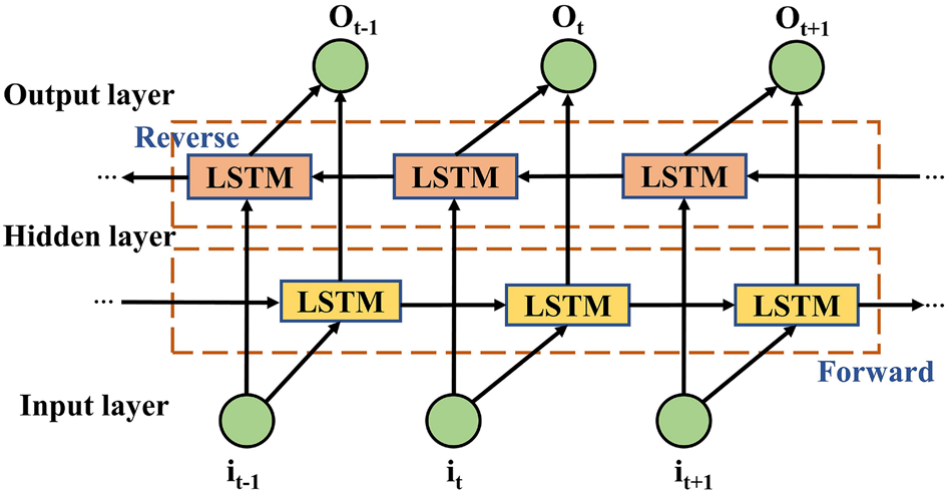
BiLSTM structure.

BiLSTM^26^ network is composed of forward and reverse LSTM neural networks, which can realise forward and reverse two LSTM training for time series, and effectively improve the comprehensiveness and completeness of feature selection. The structure of BiLSTM^27^ is shown in Fig. 2.

The output $\vec{h}_{t}$ of the forward LSTM layer in Fig. 2 is connected to the output $\mathop{h}\limits^{\leftarrow} _{t}$ of the backward LSTM layer, which is weighted and fused to obtain the final power output value $O_{t}$. The BiLSTM equation is:

6 $$ \vec{h}_{t} = \overrightarrow {{L_{LSTM} }} (h_{t - 1} ,i_{t} ),\,\,\,t = 1,2, \cdots ,n $$

7 $$ \mathop{h}\limits^{\leftarrow} _{t} = \overleftarrow {{L_{LSTM} }} (h_{t + 1} ,i_{t} ),\,\,t = 1,2, \cdots ,n $$

8 $$ O_{t} = f\left( {W_{{\vec{h}}} \vec{h}_{t} + W_{{\mathop{h}\limits^{\leftarrow}  }} \mathop{h}\limits^{\leftarrow} _{t - 1} + b_{t} } \right), $$

where $i_{t}$ is the input eigenvector; $\vec{h}_{t}$, $\mathop{h}\limits^{\leftarrow} _{t}$ forward and backward power predictions; $\overrightarrow {{L_{LSTM} }} ( \cdot )$, $\overleftarrow {{L_{LSTM} }} ( \cdot )$ is the network bidirectional computation process; $W_{{\vec{h}}}$, $W_{{\mathop{h}\limits^{\leftarrow}  }}$ is the bidirectional output connection weight matrix, $b_{t}$ is the output layer bias, and $O_{t}$ the final output power prediction of the network.

## Model construction

### Stepwise decomposition for sample construction

**Figure 3:**
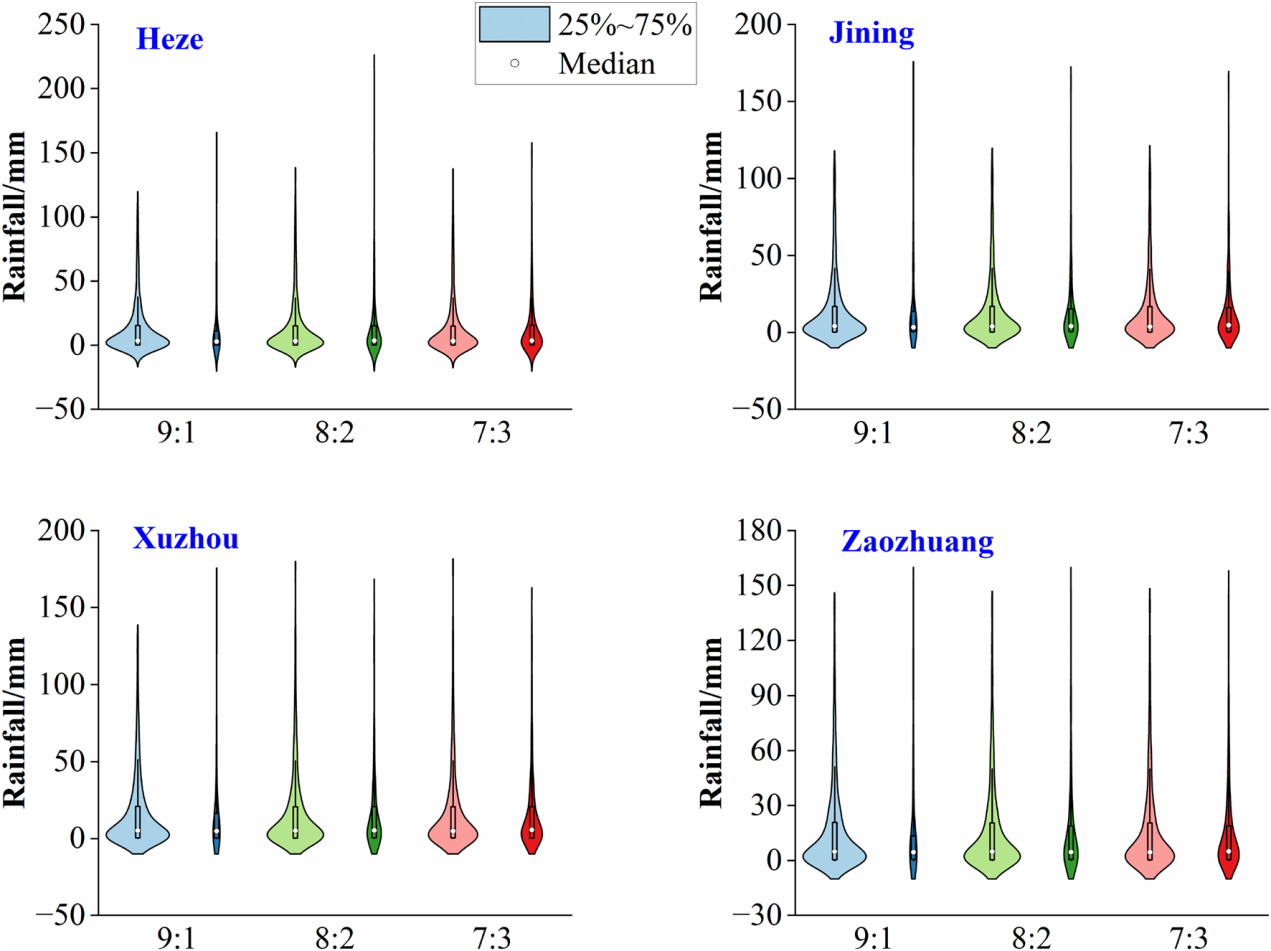
Scale violin diagram for the division of training and testing periods.

1. The rainfall sequences $(S_{1} ,S_{2} , \cdots ,S_{N} )$ is divided into training set $(S_{1} ,S_{2} , \cdots ,S_{P} )$ and test set $(S_{P + 1} ,S_{P + 2} , \cdots ,S_{N} )$. As shown in Fig. 3, the training set and test set ratios of 9:1,8:2,7:3 are considered. The violin plots illustrate the distribution of the rainfall sequences for the three allocation ratios.

According to Fig. 3, it is evident that the rainfall data for Heze, Jining, Xuzhou, and Zaozhuang exhibit relatively uniform overall distributions. The kernel density distributions of training and testing datasets are closer under both 8:2 and 7:3 allocations. By considering the closest match in terms of mean and standard deviation as indicated in Table 1, the final decision is made to set the training and testing set ratios for Heze and Zaozhuang at 7:3, while for Jining and Xuzhou, the ratios are set at 8:2.

**Table 1:** Precipitation characteristic indexes in training set and testing set.

Site	Sample	9:1		8:2		7:3	
		Mean	Standard deviation	Mean	Standard deviation	Mean	Standard deviation
Heze	Training set	13.58	23.83	13.61	23.56	13.60	23.70
	Testing set	12.61	23.36	12.97	24.68	13.19	24.00
Jining	Training set	14.54	24.06	14.59	24.20	14.66	24.66
	Testing set	14.38	26.82	14.26	24.95	14.21	23.60
Xuzhou	Training set	16.45	26.23	16.46	26.52	16.52	27.13
	Testing set	16.61	30.02	16.49	27.08	16.33	25.42
Zaozhuang	Training set	15.72	24.37	15.78	24.59	15.89	25.24
	Testing set	16.68	30.16	15.96	26.63	15.64	24.47

**Figure 4:**
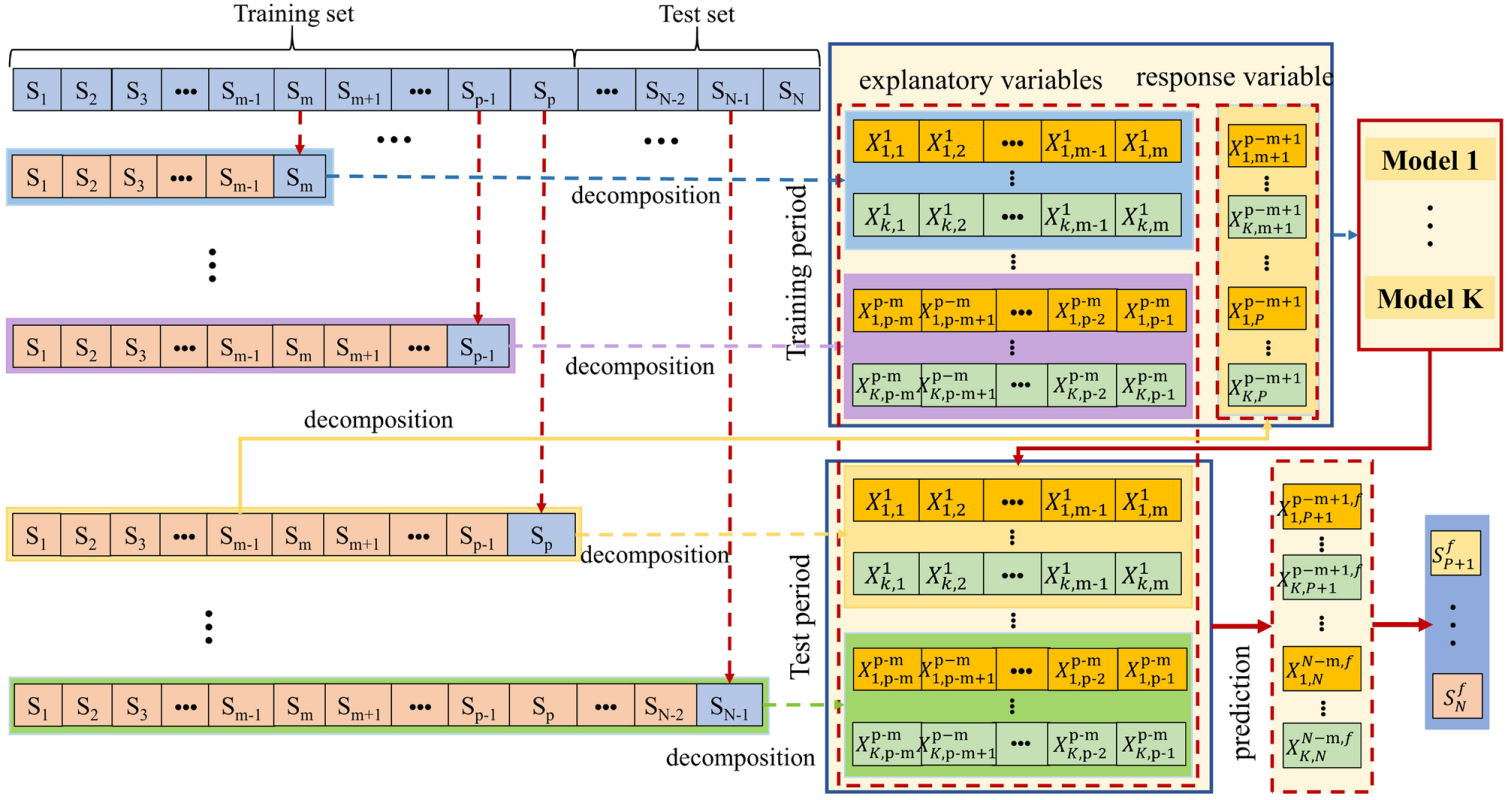
Stepwise decomposition flow chart^12^.

(2) Sequence $(S_{1} ,S_{2} , \cdots ,S_{m} )$ is decomposed into $K$ sub-sequences. Data $S_{m + 1}$ is added to sequence $(S_{1} ,S_{2} , \cdots ,S_{m} )$ to form a new sequence $(S_{1} ,S_{2} , \cdots ,S_{m} ,S_{m + 1} )$, which is then decomposed into $K$ sub-sequences. New data is sequentially added to $(S_{1} ,S_{2} , \cdots ,S_{m} )$ for decomposition.
(3) Each sequence can be decomposed into $K$ sub-sequences, from which the last m elements of each sub-sequence are extracted as explanatory variables. These explanatory variables serve as initial input data for the coupled model, which is then fine-tuned to predict the values of the response variable.
(4) The response variables of the sub-sequences obtained through decomposing sequence $(S_{1} ,S_{2} , \cdots ,S_{m} , \cdots ,S_{p - 1} ,S_{p} )$ are illustrated in Fig. 4, depicting the stepwise decomposition sampling technique^12^.

### Statistical evaluation indicators

To validate the predictive superiority of the stepwise decomposition-based VMD-IPSO-BiLSTM model, it is compared with the IPSO-BiLSTM model. Additionally, to assess the advantages of the IPSO optimization algorithm, a comparison is made between the IPSO algorithm and the conventional PSO algorithm. The predictive performance of the models is evaluated using three error metrics: mean absolute error (MAE), root mean square error (RMSE), and Nash–Sutcliffe efficiency (NSE). Smaller MAE and RMSE values and an NSE closer to 1 indicate better point prediction performance of the model. Due to the stochastic nature of the coupled model, the weekly rainfall time series data for the four cities are run 20 times, recording the results for evaluation based on error and model performance metrics. The Nash–Sutcliffe efficiency (NSE), root mean square error (RMSE), and mean absolute error (MAE) are employed as model error evaluation metrics, with the following formulas:

9 $$ RMSE = \sqrt {\frac{{\sum\limits_{i = 1}^{n} {(P\left( i \right) - P_{{}}^{*} \left( i \right))^{2} } }}{n}} , $$

10 $$ NSE = 1 - \frac{{\sum\limits_{i = 1}^{n} {\left( {P\left( i \right) - P_{{}}^{*} \left( i \right)} \right)^{2} } }}{{\sum\limits_{i = 1}^{n} {\left( {P\left( i \right) - \overline{P} } \right)^{2} } }}, $$

11 $$ MAE = \frac{1}{n}\sum\limits_{i = 1}^{n} {|P^{*} (i) - P(i)|} ,i = 1,2,...,n. $$

where $P$ is the observed value, $P^{*}$ is the predicted value, $\overline{P}$ is the mean of observed values.

## Case studies

### Data sources

**Figure 5:**
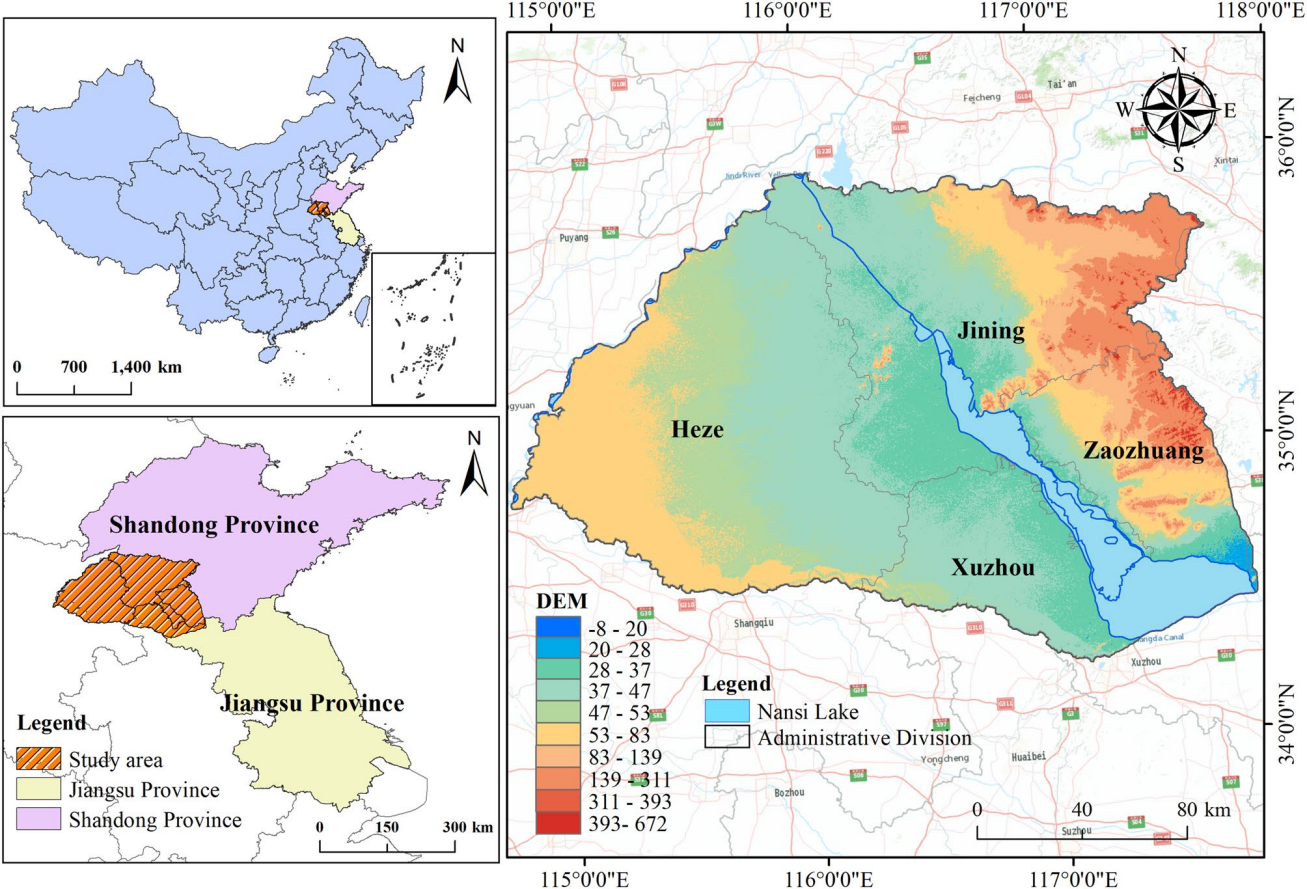
Research situation of Nansi Lake basin.

Nansi Lake, located in Shandong Province, China, is the largest lake in the province and one of the largest freshwater lakes in China. With a basin area of 31,700 km^2^, it serves multiple ecological functions and serves as a crucial reservoir for the South-to-North Water Diversion Project, playing a significant role in water diversion and storage. The average annual precipitation in the South Four Lakes basin is 731 mm, with a total storage capacity of 4.731 billion m3 and an average annual available water volume of 1.273 billion m3. An overview of the study area is presented in Fig. 5. The map in Fig. 5 was created using the ArcGIS software version10.8, available at http://www.esri.com/software/arcgis.

**Figure 6:**
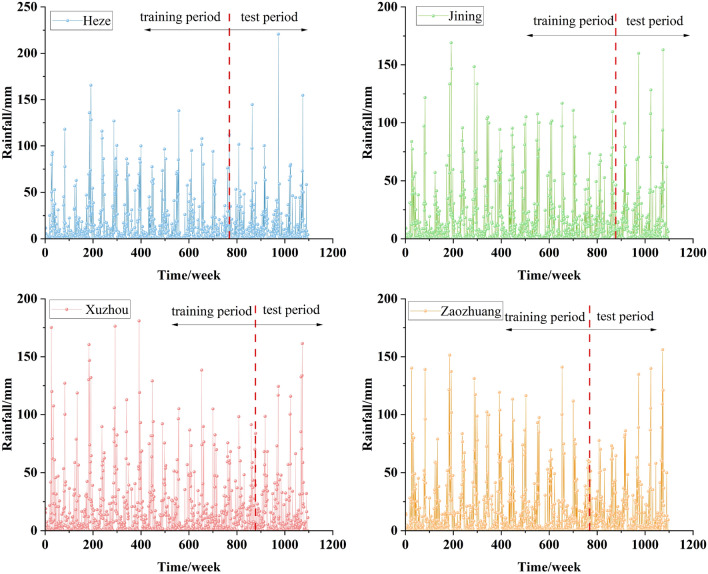
Weekly rainfall series of four cities in the South Four Lakes basin.

The Nansi Lake Basin encompasses the cities of Heze, Jining, Xuzhou, and Zaozhuang. Daily rainfall data for these four cities were obtained from the National Meteorological Science Data Center (https://data.cma.cn/). The 7671 daily rainfall data points spanning from January 1, 2000, to December 31, 2020, were organized into 1096 sets of weekly rainfall data. The data was split according to the allocation ratios for the training and testing periods. For Heze and Zaozhuang, the training period spans from January 1, 2000, to September 26, 2014, and the testing period from September 27, 2014, to December 31, 2020. For Jining and Xuzhou, the training period extends from January 1, 2000, to October 28, 2016, and the testing period from October 29, 2016, to December 31, 2020. Please refer to Fig. 6 for details.

### Model training

The BiLSTM model features a bidirectional recurrent neural network with input and output layers, as well as hidden layers. The Adam optimizer is selected to update the model parameters. The IPSO optimization model is configured with a population size of 50, an inertia weight of 0.7, and individual learning factors $C_{1}$ and social learning factors $C_{2}$ as shown in Table 2.

**Table 2:** Table of optimal parameter combinations for each model.

Cities	Model	N	L	$H_{1}$	$H_{2}$	$C_{1}$	$C_{1}$
Heze	PSO-BiLSTM	210	0.009	83	40	1.5	1.6
	IPSO-BiLSTM	155	0.006	50	59	1.6	1.6
	VMD-PSO-BiLSTM	299	0.008	85	45	1.8	1.9
	VMD-IPSO-BiLSTM	330	0.009	65	30	1.6	1.7
Jining	PSO-BiLSTM	240	0.007	82	18	1.6	1.7
	IPSO-BiLSTM	170	0.005	56	44	1.5	1.6
	VMD-PSO-BiLSTM	200	0.007	80	40	1.8	1.5
	VMD-IPSO-BiLSTM	320	0.006	60	47	1.8	1.8
Xuzhou	PSO-BiLSTM	240	0.008	76	93	1.8	1.8
	IPSO-BiLSTM	230	0.007	60	50	1.9	1.6
	VMD-PSO-BiLSTM	230	0.005	87	69	1.9	1.9
	VMD-IPSO-BiLSTM	369	0.007	66	36	1.5	1.7
Zaozhuang	PSO-BiLSTM	220	0.007	80	26	1.7	1.7
	IPSO-BiLSTM	190	0.007	65	50	1.9	1.5
	VMD-PSO-BiLSTM	250	0.008	90	50	1.8	1.6
	VMD-IPSO-BiLSTM	358	0.008	69	50	1.8	1.8

Addressing issues such as manual parameter tuning and slow convergence speed in the BiLSTM neural network predictive model, the particle swarm optimization (PSO) algorithm is employed to optimize the iteration count (N), learning rate (L), the number of nodes in the first hidden layer ($H_{1}$), and the number of nodes in the second hidden layer ($H_{2}$). The optimal parameter combination is ultimately obtained, as presented in Table 2.

### Model prediction results

**Figure 7:**
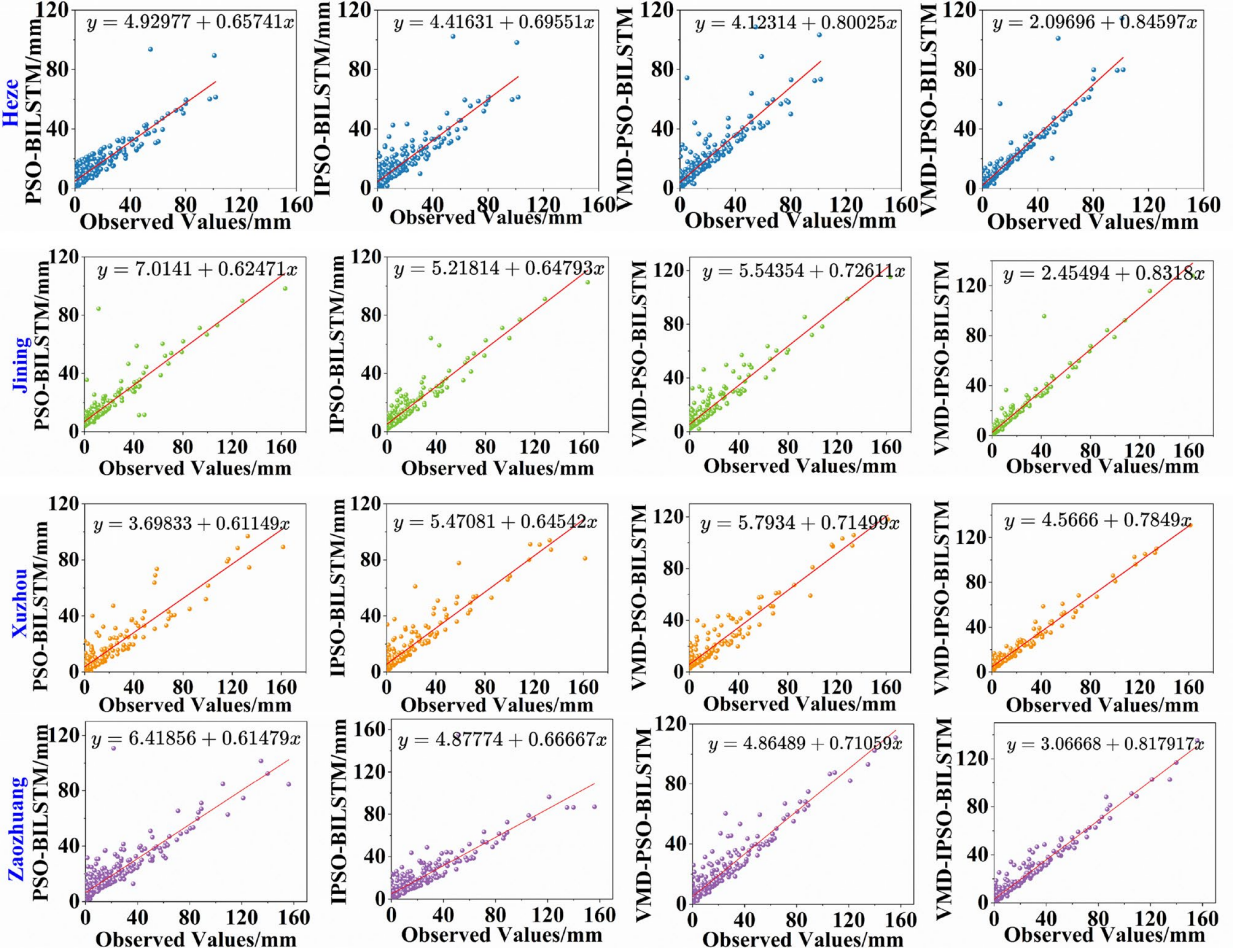
Scatterplot of rainfall series prediction results for each city.

To better analyze the predictive performance of each model during the testing period, Fig. 7 presents a scatter plot of observed values against predicted values. The red line in the figure represents the linear fitting line for observed and predicted values. A comparison between PSO-BiLSTM and IPSO-BiLSTM reveals that the scatter distribution of IPSO-BiLSTM is more compact, and the slope of the linear fitting line is closer to 1 during the testing period for each city. Additionally, comparing the predictive results of VMD-PSO-BiLSTM and VMD-IPSO-BiLSTM shows that the slopes of VMD-IPSO-BiLSTM are all greater than 0.78, indicating a more compact scatter distribution. This suggests that the IPSO-optimized VMD-BiLSTM model outperforms the VMD-BiLSTM model optimized with conventional PSO. The IPSO algorithm demonstrates its ability to enhance the predictive accuracy of both individual models and decomposition ensemble models. In comparison to the two individual models (PSO-BiLSTM and IPSO-BiLSTM), the hybrid models (VMD-PSO-BiLSTM and VMD-IPSO-BiLSTM) exhibit superior performance in rainfall prediction across the four cities.

### Multi-model comparison

Table 3 presents the model accuracy evaluation results during the training and testing periods for the IPSO-optimized models. During the training period, PSO-BiLSTM, IPSO-BiLSTM, and VMD-PSO-BiLSTM demonstrated optimal simulation results in Heze, while VMD-IPSO-BiLSTM exhibited superior performance in simulating Zaozhuang. In the testing period, VMD-IPSO-BiLSTM outperformed other models in terms of performance across all cities.

**Table 3:** Evaluation results of each model in the training and testing periods.

Cities	Model		PSO-BiLSTM	IPSO-BiLSTM	VMD-PSO-BiLSTM	VMD-IPSO-BiLSTM
Heze	Training period	MAE	5.42	5.27	4.63	2.99
		RMSE	9.40	8.86	8.29	5.76
		NSE	0.67	0.73	0.81	0.92
	Testing period	MAE	6.30	6.13	5.32	3.44
		RMSE	10.94	10.30	9.54	6.63
		NSE	0.45	0.59	0.73	0.88
Jining	Training period	MAE	6.44	5.63	5.27	2.74
		RMSE	10.06	8.61	7.98	5.09
		NSE	0.63	0.73	0.81	0.94
	Testing period	MAE	7.27	6.32	5.88	3.43
		RMSE	11.37	9.66	8.91	6.36
		NSE	0.50	0.62	0.75	0.90
Xuzhou	Training period	MAE	6.88	6.46	7.35	4.48
		RMSE	11.68	10.95	10.38	6.11
		NSE	0.61	0.68	0.73	0.92
	Testing period	MAE	7.65	6.97	6.88	5.12
		RMSE	12.99	11.81	9.72	6.98
		NSE	0.43	0.60	0.77	0.89
Zaozhuang	Training period	MAE	6.72	6.15	5.12	3.11
		RMSE	10.86	10.83	7.86	4.79
		NSE	0.62	0.67	0.83	0.95
	Testing period	MAE	7.57	6.76	5.93	3.94
		RMSE	12.23	11.91	9.10	6.10
		NSE	0.48	0.58	0.75	0.91

As shown in Table 3. Comparing the prediction results of PSO-BiLSTM and IPSO-BiLSTM for each city in the test period, it can be seen that the MAE and RMSE values of the IPSO-BiLSTM model are smaller, and the NSE value is closer to 1. The MAE value decreases from 6.30 to 7.65 to 6.13 to 6.97, and the RMSE decreases from 10.94 to 12.99 to 9.66 to 11.91. NSE values improved from 0.43 ~ 0.50 to 0.309 ~ 0.630, indicating that the performance of the IPSO-optimised BiLSTM model is better than that of the ordinary PSO-optimised BiLSTM model. The IPSO algorithm can improve the model overfitting problem, and thus improve the predictive performance of the model.

Comparing the predictions of IPSO-BiLSTM and VMD-IPSO-BiLSTM, the VMD-IPSO-BiLSTM model demonstrates the most significant reduction in MAE in Jinan, reaching 45.73%. The RMSE value sees the most noticeable decrease in Zaozhuang, reaching 48.78%. This implies that the stepwise decomposition technique based on VMD significantly improves the performance of the coupled prediction model, leading to a minimum 45% increase in NSE values for rainfall testing across all cities.

**Figure 8:**
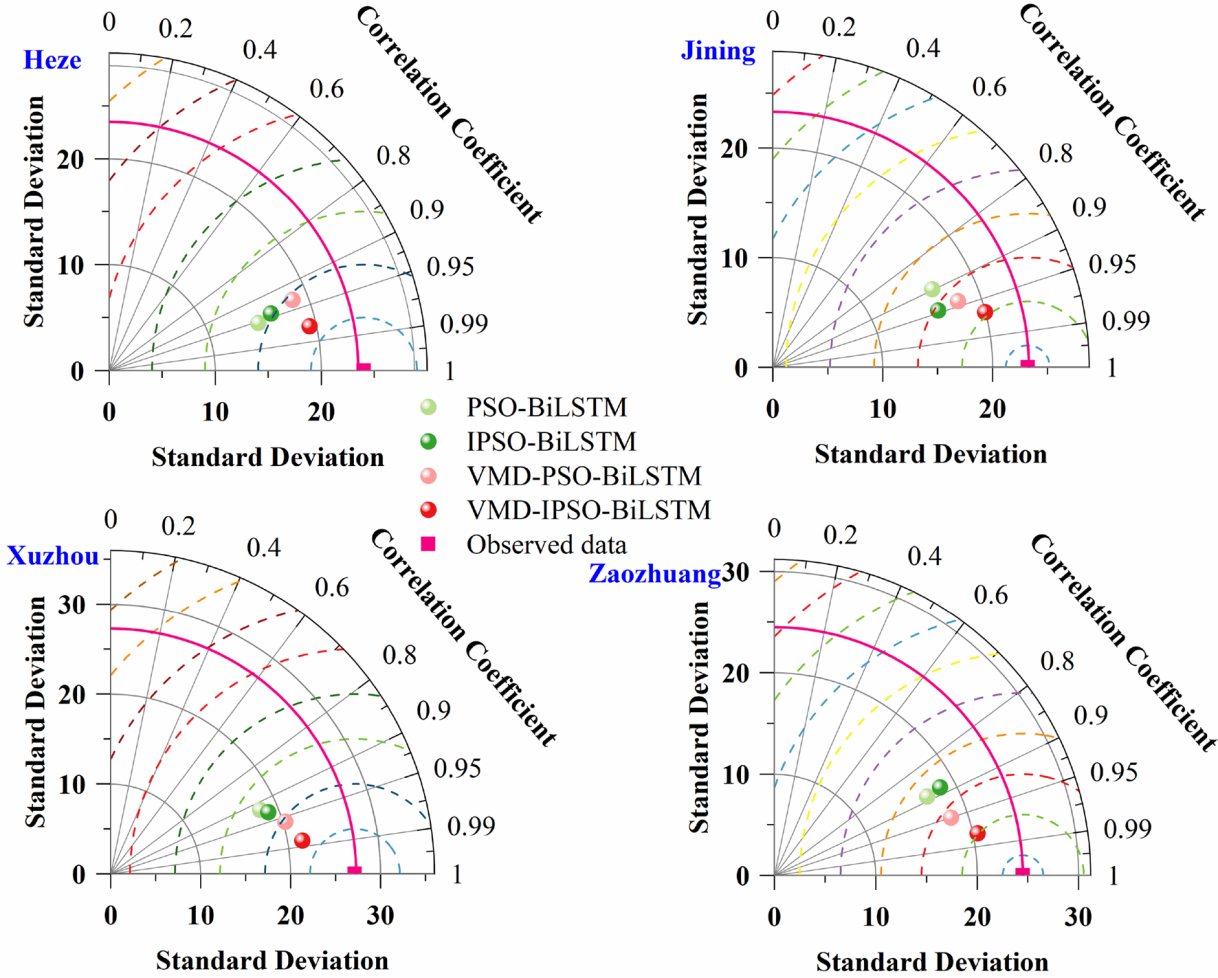
Taylor diagram comparing the prediction performance of multiple models.

To assess model accuracy, Taylor diagrams were employed, as depicted in Fig. 8. The correlation coefficients and standard deviations of different models for the four cities were compared. Concerning the correlation coefficients, the simulation results for Heze, Jinan, and Xuzhou fall within the range of 0.9 to 0.99, while Zaozhuang's simulation results range from 0.8 to 0.99. Regarding standard deviations, VMD-IPSO-BiLSTM closely approximates observed values in terms of standard deviations across all four cities.

## Discussion

The traditional decomposition-prediction coupling model technique, when applied to decompose time series data, may inadvertently introduce some testing period data into the training period. Constructing a decomposition ensemble model can result in "false" high-accuracy prediction outcomes, making it challenging for the model to meet the demands of practical forecasting work^19,20^. The VMD-IPSO-BiLSTM model, based on the stepwise decomposition technique, sequentially appends new data $S_{m + 1}$ to the existing sequence segment $(S_{1} ,S_{2} , \cdots ,S_{m} )$ for decomposition, gradually extends the sequence segment, and extracts corresponding explanatory samples.

Compared to previous VMD-BiLSTM approaches^28,29^, the model performance is significantly enhanced by optimizing model parameters through the IPSO optimization algorithm. Notably, the VMD-IPSO-BiLSTM model does not utilize testing period data during model training. However, in contrast to optimized prediction models based on traditional decomposition methods^30^, the predictive accuracy of the stepwise decomposition model is relatively lower. Therefore, further considerations are needed to improve model accuracy in the future.

## Conclusion

To enhance the accuracy of rainfall prediction models, this paper introduces a novel VMD-IPSO-BiLSTM stepwise decomposition ensemble model. Comparative analysis of the prediction results with the PSO-BiLSTM, IPSO-BiLSTM, and VMD-PSO-BiLSTM models reveals the following research findings:

1. Compared with PSO-BiLSTM, the MAE value of IPSO-BiLSTM model decreases from 6.30 to 7.65 to 6.13 to 6.97, the RMSE decreases from 10.94 to 12.99 to 9.66 to 11.91, and the NSE value improves from 0.43 to 0.50 to 0.309 to 0.630, which indicates that the IPSO-optimised BiLSTM model parameters with better performance.
2. Contrast between IPSO-BiLSTM and VMD-IPSO-BiLSTM predictions reveals that the VMD-IPSO-BiLSTM model achieves the most substantial reduction in MAE in Jinan, reaching 45.73%. RMSE values experience the most notable decrease in Zaozhuang at 48.78%. This highlights the significant improvement in the performance of the coupled prediction model based on VMD's stepwise decomposition technique, leading to a minimum 45% increase in NSE values during the rainfall testing period across various cities.
3. VMD-IPSO-BiLSTM effectively addresses the issue of erroneously using validation period forecast factor numbers in traditional decomposition ensemble prediction models. NSE values during the testing period exceed 0.88 in all cities, indicating higher predictive accuracy. This model provides valuable reference for the correct establishment of decomposition ensemble rainfall prediction models and serves as a basis for practical forecasting of non-stationary and non-linear rainfall sequences.

## Data Availability

Data and materials are available from the corresponding author upon request.

## Code availability

The code mentioned in the article is stored in a public repository on GitHub and is executed using MATLAB 2020 software. VMD: https://github.com/vrcarva/vmdpy. BILSTM: https://github.com/albertwy/BiLSTM. PSO: https://github.com/EddyGao/PSO.
